# Promoting Public Health Through State Cancer Control Plans: A Review of Capacity and Sustainability

**DOI:** 10.3389/fpubh.2015.00040

**Published:** 2015-03-18

**Authors:** Marcia G. Ory, Brigid Sanner, Deborah Vollmer Dahlke, Cathy L. Melvin

**Affiliations:** ^1^Department of Health Promotion and Community Health Sciences, Texas A&M Health Science Center School of Public Health, College Station, TX, USA; ^2^Sanner & Company, Broomfield, CO, USA; ^3^Medical University of South Carolina, Charleston, SC, USA

**Keywords:** cancer prevention and control, capacity, sustainability, state plans, evidence-based practices

## Abstract

The Centers for Disease Prevention and Control’s National Comprehensive Cancer Control (CCC) Program oversee CCC programs designed to develop and implement CCC plans via CCC coalitions, alliances, or consortia of program stakeholders. We reviewed 40 up-to-date plans for states and the District of Columbia in order to assess how capacity building and sustainability, two evidence-based practices necessary for organizational readiness, positive growth, and maintenance are addressed. We employed an electronic key word search, supplemented by full text reviews of each plan to complete a content analysis of the CCC plans. Capacity is explicitly addressed in just over half of the plans (53%), generally from a conceptual point of view, with few specifics as to how capacity will be developed or enhanced. Roles and responsibilities, timelines for action, and measurements for evaluation of capacity building are infrequently mentioned. Almost all (92%) of the 40 up-to-date plans address sustainability on at least a cursory level, through efforts aimed at funding or seeking funding, policy initiatives, and/or partnership development. However, few details as to how these strategies will be implemented are found in the plans. We present the Texas plan as a case study offering detailed insight into how one plan incorporated capacity building and sustainability into its development and implementation. Training, technical assistance, templates, and tools may help CCC coalition members address capacity and sustainability in future planning efforts and assure the inclusion of capacity building and sustainability approaches in CCC plans at the state, tribal, territorial, and jurisdiction levels.

## Introduction

The Centers for Disease Prevention and Control’s (CDC) National Comprehensive Cancer Control Program (NCCPC) fund 65 awardees (states, territories, Pacific Island jurisdictions, and tribes/tribal organizations) in 69 Comprehensive Cancer Control (CCC) programs to develop and implement CCC plans via CCC coalitions, alliances, or consortia of program stakeholders (1, 2). CCC is a process through which communities and partner organizations pool resources to reduce the burden of cancer by reducing cancer risk, finding cancers earlier, improving treatments, increasing the number of people who survive cancer, and improving the quality of life for cancer survivors (2). CCC plans are intended to guide community and state-wide activities in cancer prevention and control in states, territories, tribes and tribal organizations, and Pacific Island jurisdictions. The plans are intended to be data-driven, evidence-based blueprints for action, and generally cover a 5-year timeframe. In the fifth year, different processes are used to revise and update CCC plans. CCC plans serve as written documentation of the burden of cancer and offer blueprints for coordinated action – ideally laying out measurable objectives and specifying which organizations will be responsible for supporting specific strategies to meet those objectives (3).

**Figure 1:**
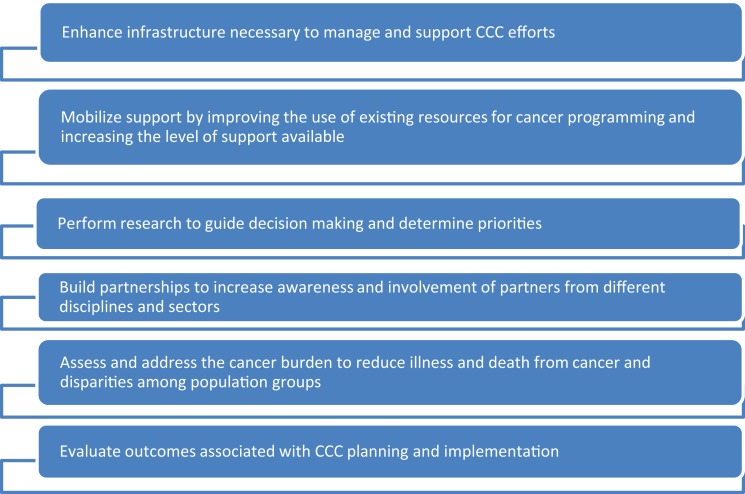
**Building blocks of CCCs**.

Centers for Disease Prevention and Control’s NCCPC provide support for CCC Coalitions to develop and implement CCC plans that include approaches to enhance the basic building blocks of CCC (see Figure 1).

Chief among these building blocks are community capacity and sustainability, two constructs considered as essential for achieving long-term success in designing, implementing, and disseminating cancer-related public health practices and policies. Despite their importance, little is known about the extent to which community capacity building and sustainability constructs are addressed in current cancer control plans. The purpose of this article is to offer a review of state plans, including the District of Columbia, and document where and in what manner these two constructs are explicitly addressed. Based on a review of select state plans, recommendations are offered that are designed to assist states to more effectively address community capacity building and sustainability as plans are updated and revised.

This review was compiled in 2012–2014 under the direction of two member-centers of the 2009–2015 Cancer Prevention and Control Research Network (CPCRN): Texas A&M Health Science Center and the University of North Carolina, Chapel Hill. During this period, both institutions were CDC Prevention Research Centers (PRC). The PRCs are CDC’s flagship programs for preventing and controlling chronic diseases. CPCRN provides an infrastructure for applying relevant research to local cancer prevention and control needs. Its members conduct community-based participatory cancer research across its 10 network centers, crossing academic affiliations and geographic boundaries.

## Materials and Methods

### Determining status of plans

An online review of 51 CCC Plans (all states and the District of Columbia) was conducted, utilizing plans posted on the Cancer Control P.L.A.N.E.T.^1^ portal. The portal provides access to resources that can assist in: (1) assessing the cancer and/or risk factor burden within a given state; (2) identifying potential partners that may already be working with high-risk populations; (3) understanding the current research findings and recommendations; (4) accessing and downloading evidence-based programs and products; and (5) finding guidelines for planning and evaluation (4).

If a state’s plan as posted on the portal was out-of-date, a subsequent search was conducted using state department of health/public health websites and state cancer consortium websites to attempt to identify an up-to-date document for review. Initial scans done in 2012 found 31 of the 51 plans (61%) were current. A follow-up scan done in 2014 found 40 of 51 (78%) up-to-date plans, including 10 plans that were out-of-date in 2012, but updated at the time of the 2014 scan. Nine state plans were out-of-date in 2012 and remained out-of-date in 2014. Two state plans were current in 2012, but outdated in the 2014 scan. See Table 1 for details of the status of state and the District of Columbia’s plans at these two time points.

**Table 1:** **Status of state cancer control plans**.

State	2012 scan plan in effect until	2014 scan plan in effect until
Alabama	2015	2015
Alaska	2010	2010
Arizona	Out-of-date	Out-of-date
Arkansas	Out-of-date	Out-of-date
California	2004	2015. Available on the California Department of Public Health website. The 2004 document is still posted on Cancer Control P.L.A.N.E.T.
Colorado	Out-of-date	2015
Connecticut	2013	2017. Available on the Connecticut Cancer Partnership website. The 2013 document is still posted on Cancer Control P.L.A.N.E.T.
Delaware	2011	2016. Available on the Delaware Cancer consortium website. The 2011 document is still posted on Cancer Control P.L.A.N.E.T.
District of Columbia	2010	2018
Florida	No end date – plan appears current	No end date – plan appears current
Georgia	2012	2019. Available on the Georgia Department of Public Health website. The 2012 document is still posted on Cancer Control P.L.A.N.E.T.
Hawaii	2009	2015. Available on the Hawaii state health website. The 2009 document is still posted on Cancer Control P.L.A.N.E.T.
Idaho	2015	2015
Illinois	2010	2015
Indiana	2014	2014
Iowa	2017	2017
Kansas	Out-of-date	2016
Kentucky	No end date – plan appears current	No end date – plan appears current
Louisiana	2015	2015
Maine	2015	2015
Maryland	Out-of-date	2015
Massachusetts	2016	2016
Michigan	2015	2015
Minnesota	2016	2016
Mississippi	2011	2011
Missouri	2015	2015
Montana	2016	2016
Nebraska	2016	2016
Nevada	2015	2015
New Hampshire	2014	2014
New Jersey	2012	2012
New Mexico	2011	2017. Available on the New Mexico Cancer Council website. The 2011 document is still posted on Cancer Control P.L.A.N.E.T.
New York	2010	2017
North Carolina	2008	2008
North Dakota	2016	2016
Ohio	2014	2014
Oklahoma	2010	2010
Oregon	2010	2010. Addendums to parts of the plan were posted on the Oregon Health authority website September 2012
Pennsylvania	2003	Draft framework for a 2018 plan is available at www.pacancersummit2013.pitt.edu/plan.html
Rhode Island	2012	2018. Available on the Health Rhode island website. The 2012 document is still posted on Cancer Control P.L.A.N.E.T.
South Carolina	2015	2015
South Dakota	2015	2015
Tennessee	2012	2017. Available on the Tennessee state health department website. The 2012 document is still posted on Cancer Control P.L.A.N.E.T.
Texas	2005	2016
Utah	2015	2015
Vermont	2015	2015
Virginia	2012	2017. Available on the Virginia Department of Health Website. The 2012 document is still posted on Cancer Control P.L.A.N.E.T.
Washington	2013	2013
West Virginia	Out-of-date	Out-of-date
Wisconsin	2015	2015
Wyoming	2015	2015

### Conceptual framework and definitions

This review focused on the specifics of how each plan addressed the community capacity building and sustainability constructs, both of which are critical if planned activities are to be effectively implemented and maintained throughout the plan’s timeline. The National Council of Non-profits notes that distinct capacity-building projects such as identifying a communications strategy, improving volunteer recruitment, developing a leadership succession plan, identifying more efficient uses of technology, and engaging in collaborations with community partners all contribute to building capacity (5).

Other definitions of capacity include R. J. Chaskin’s description in the Urban Affairs Review, “Community capacity building is the interaction of human capital, organizational resources, and social capital existing within a given community that can be leveraged to solve collective problems, and improve or maintain the well-being of that community” (6). Goodman et al. suggest that capacity is a “potential state” representing ability to act (7). Foster-Fishman et al. conclude from their review of the literature that capacity must emerge at four levels for community-based coalitions: (a) within their members, (b) within their relationships, (c) within their organizations (including funding and staffing), and (d) within the programs they sponsor (8).

The Aspen Institute’s *Measuring Community Capacity Building* similarly notes that community capacity building is viewed as building durable resources within a community or organization. It involves a combined influence of a community’s commitment, resources, and skills that can be deployed to build on community strengths and address community problems and opportunities (9).

*Measuring Community Capacity Building* outlines the elements of *general capacity* as: leadership, participation and opportunities for participation, resources, connections among people and organizations, connections with outside communities and institutions, sense of community, norms and values, commitment, community power, and community knowledge and skills. *Measuring Community Capacity Building* outlines the elements of *organizational capacity* as: leadership, organizational structure/management style, organizational climate, resource availability, staff capacity, and external relationships.

If one thinks of capacity as identifying, building and leveraging resources, sustainability assures that programs can operate long-term. “Sustainability is the continued use of program components and activities for the continued achievement of desirable program and population outcomes. Other terms in this domain include continuation, confirmation, maintenance, durability, continuance, and institutionalization. Nuanced differences exist among these terms, but they all usually refer to the continued use of program components and activities beyond their initial funding period, and sometimes to continuation of desired intended outcomes, which we are calling “sustainability” (10). Elaborating on the review methods previously noted, to consider sustainability of efforts, each CCC plan was reviewed to see if and how it addressed:

∙ the capacity to maintain service coverage at a level that will provide continuing control of a health problem (11);
∙ the capacity of a project to continue to deliver its intended benefits over a long period of time (12);
∙ the delivery of an appropriate level of benefits for an extended period of time after major financial, managerial, and technical assistance from an external donor is terminated (13).

### Systematic review of all plans

Each up-to-date state plan, downloaded from the Cancer Control P.L.A.N.E.T. website, state department of health, or CCC Coalition website was reviewed using an electronic search for specific terms agreed upon by authors and based on their expertise in cancer prevention and control and public health concepts. Search terms were: capacity; community capacity; building; training; sustain/sustainability/sustaining; funding; and support.

If these key words were found in a CCC plan, a reviewer read the plan to determine, from a subjective standpoint, if and how community capacity building and/or sustainability were being pragmatically addressed.

### Case study

In addition to the review of the up-to-date plans, an in-depth review of one state plan that of Texas, was conducted and informed by a telephone interview with a member of the plan’s working group. The objective of this case study was to provide a more detailed snapshot of how a plan that addressed sustainability and capacity-building constructs was developed and implemented. The Texas plan was selected because it built on previously developed CCC plans from that state, was developed by a CCC Coalition representing a large area with significant diversity, and offered several concrete examples of an effective approach to community capacity building and sustainability. As with the overviews of the other plans, this review is intended to provide state planners with insight that might be useful as future plans are created, updated, or revised.

## Results

### Overview of plans

A general review indicated similarities and differences across current plans. All 40 plans up-to-date at the time of the 2014 scan were developed by a collaborative (e.g., coalition, alliance, consortium) of organizations that was convened regularly (at least annually). In some cases, such as Virginia, collaboratives met more frequently. Monitoring and updating each plan seem to be the responsibility (either implicitly stated or implied) of each state collaborative.

The majority of the plans were developed through a committee process, often with sections developed by work groups (such as cancer prevention, clinical trials, and survivorship) whose members have specific expertise related to the specific section of the CCC plan. These sections are combined into the comprehensive plan.

The organization of the plans varies across states with some plans structured to address the cancer continuum as well as site-specific cancers and others, focused solely on site-specific cancers. Plans varied widely in their level of detail, with some including extensive state cancer data, which can serve as a resource for reference by those interested in cancer statistics within a particular state while others simply summarized goals, objectives, and strategies.

### Community capacity

Capacity is referred to in 21 of the 40 up-to-date plans (53%) although it is generally addressed only from a conceptual point of view, with few specifics as to how capacity will be developed or enhanced, roles and responsibilities specified, timelines for action enumerated, or measurements for evaluation of capacity building delineated. Table 2 summarizes the sections in which the 21 state plans addressed capacity, even if only in a conceptual manner.

**Table 2:** **Capacity concept addressed in some objectives or strategies of the following plans**.

State	Section(s) where capacity is referenced
Alabama	∙ Evaluation
	∙ Advocacy
California	∙ Health disparities
	∙ Surveillance
Colorado	∙ Health equity
	∙ Health care systems
	∙ Evaluation and research
Connecticut	∙ Screening and early detection
	∙ Evaluation
District of Columbia	∙ Executive summary
	∙ Introduction
	∙ Breast cancer
	∙ Health equity
	∙ Prostate cancer
	∙ Budget
Florida	∙ Infrastructure component: capacity
Indiana	∙ Early detection: screening
Iowa	∙ Quality of life: survivorship
Kentucky	∙ Early detection and screening
Massachusetts	∙ Colorectal screening
Michigan	∙ Guiding principles
	∙ Implementation evaluation
Missouri	∙ Colorectal screening
Nebraska	∙ Introduction (memorandum of understanding)
	∙ Access to care
New Mexico	∙ Coordination, implementation, evaluation
North Dakota	∙ Screening/early detection (colorectal)
	∙ Health equity
	∙ Evaluation
South Carolina	∙ Cross-cutting goal on capacity
South Dakota	∙ Quality of life
Tennessee	∙ Childhood/adolescent cancers
Texas	∙ Overarching principle
	∙ Colorectal screening
	∙ Awareness (community)
Utah	∙ Strategic planning model
	∙ Capacity building
Wyoming	∙ Early detection: breast, cervical, and colorectal cancers
	∙ Childhood cancer: psychological and physical health

### Case examples of community capacity

Despite the widespread lack of details that consider “who,” “how,” and “when,” a number of plans did consider capacity building with some detail. Noteworthy examples include:

∙ District of Columbia: this plan takes the step of providing a budget breakdown for plan implementation, which includes a $75,000 line-item for coalition member technical assistance and capacity building.
∙ Florida: the Florida plan includes an overarching component on capacity, which lists 10 specific strategies for capacity building.
∙ Michigan: the importance of capacity is highlighted in the plan’s guiding principles: “Cancer control priorities should be established based upon capacity for collaborative partnerships among public health agencies, private organizations, cancer centers, and all other interested agencies and organizations to carry out recommended cancer control activities.”
∙ Nebraska: a memorandum of understanding developed between the Nebraska Cancer Coalition and the Nebraska CCC Program is featured in the plan’s introduction. This document outlines the relationship and respective roles and responsibilities of the two entities as well as joint roles and responsibilities. The intention is for the relationship to be seamless while at the same time enhancing Nebraska’s capacity to maintain a state-wide partnership, develop, and implement a state cancer plan and meet the overall goal of reducing the burden of cancer in Nebraska.
∙ South Carolina: a specific capacity building goal is included in South Carolina’s plan: “To increase the effectiveness of our cancer prevention and control activities by ensuring adequate resources such as high quality data, funds, and an educated work-force, as well as effective allocation of these resources.” Contained within this cross-cutting goal are goals and strategies related to human resources, information services, and financial resources.
∙ Texas: five principles are outlined in the plan’s introduction, which includes defining measurable and realistic targets based on review of a baseline and trend data for cancer prevention and control key measures, with consideration of factors such as available resources, barriers, and capacity for implementation of strategic actions.
∙ Utah: a strategic prevention framework model addressing capacity building and partnership creation is included in the plan, which states, “Creating and implementing a comprehensive strategic plan to control cancer requires diverse perspectives and resources. Health care providers, oncology specialists, researchers, cancer survivors, advocates, public health professionals, insurers, and employers all need to be involved in the process. The makeup of each workgroup, as well as the state-wide coalition, should reflect multiple perspectives.”
∙ Additionally, Utah’s model raises the following questions based on the Substance Abuse and Mental Health Services Administration’s (SAMHSA) five-step public health planning process: (1) assess population needs, resources required, and readiness to act; (2) build capacity; (3) develop a comprehensive plan; (4) implement evidence-based programs; and (5) monitor implementation, evaluate effectiveness, sustain effective activities, and improve or replace those that fail (14). Other key activities in this step of the strategic prevention framework include educating and training stakeholders, organizing networks, gathering resources, and building leadership.
∙ Vermont: the Vermont plan also references SAMHSA’s public health planning model just noted, with step 2 relating to capacity building.

### Overview of sustainability results

Almost all [37 (92%)] of the 40 up-to-date plans address sustainability at least on a cursory level, through seeking funding, developing policy initiatives, and/or pursuing partnership development. Table 3 summarizes many of the areas related to sustainability.

**Table 3:** **Sustainability concept addressed in some objectives or strategies of the following plans**.

State	Section(s) where sustainability is referenced
Alabama	Funding cancer control initiatives: work with policy makers and partners to ensure that budgetary costs of cancer control in Alabama will be based on expected improvements in long-term societal costs. To ensure sustainability, reach out to new partners
	Tobacco: indoor clean air policy. Tobacco excise tax increases
California	Health disparities: provide capacity building, technical assistance, and resources to sustain local efforts; expand federal and state funding for services related to reducing disparities, especially in areas identified as gaps; assist local coalitions in identifying outside private funding sources
	Prostate cancer: support funding for research to identify better screening tools and develop treatment options
	Advocacy: educate public, healthcare professionals, and policy makers to garner support for funding; participate in media advocacy efforts
	Breast cancer: support research and grants for clinical trials, state and federal funding for access to screening and diagnosis, private funding, and grants for uninsured and under-insured people
	Cervical cancer: develop and promote clinical standards and professional education materials that promote follow-up and treatment for abnormal screening tests. Support funding for state and federally funded programs
	Advocate for funding of melanoma research
	Increase National Cancer Institute funding for ovarian cancer research in California by 38%
Colorado	Sustain colorectal screening funding for un- and under-insured
	Support the development of a sustainable mechanism for collecting the data to monitor sun safety guidelines in schools
	Increase funding to expand and sustain survivor education
Connecticut	Partners charged to work collaboratively to leverage support for implementation, using plan as a guide for resource allocation
Delaware	Monitor draft policies related to clean indoor air
	Support funding for physical activity
District of Columbia	A cross-cutting theme throughout the plan is to identify and develop public and private funding sources
	Sustain and expand data collection
Florida	Infrastructure: secure funds for cancer control
	Advocate for research advances
	Overarching strategy: policy and legislation
Georgia	Plan contains a section on sustainability which includes funding and work-force needs. The tobacco settlement is referenced as a key funding source
Hawaii	Advocate for equal access to, and adequate resources for screening and care
	Establish policies for prevention and early detection, affordable care, and support for survivors and families
Illinois	Implementation section addresses sustainability
	Advocate for increased funding for screening, survivorship, data surveillance, research, and clinical trials
	Tobacco policy
Indiana	Funding for tobacco education, screening for low-income individuals, Indiana Behavioral Risk Factor Surveillance System (BRFSS), and the Comprehensive Cancer Control Program
Iowa	Build and sustain coalitions
Kansas	Advocate for funding for school physical activity programs, fruit and vegetable consumption in schools, early detection
Kentucky	Sustained infrastructure for tobacco initiatives, funding for tobacco prevention and control, advocate for colon cancer screening
Louisiana	Identify funding sources for cancer control programs
Maine	Pursue sustainable funding and legislative support for Maine CCC Plan
	Use program evaluation framework to assure sustainability of plan
Maryland	Tobacco policies
Massachusetts	Develop and sustain collaborations to reduce cancer-related health disparities and promote health equity
	Create and sustain a cancer policy and legislative agenda that supports projects across the continuum
	Create and sustain environments that support prevention
Michigan	Coalition guiding principles include sustainability
Minnesota	Advocate for sustained funding for programs related to healthy eating, physical activity, healthy weight, tobacco control
	Establish consistent and reliable funding for tobacco control
Missouri	Advocate for funding to tobacco initiatives and fitness initiatives
Montana	Advocate for tobacco policies and childhood cancer survivors’ quality of life services
Nebraska	Sustain detection and screening programs
	Advocate for increased taxes on tobacco products
Nevada	Advocate for research funding
New Hampshire	Support tobacco prevention program funding
	Enhance existing and develop new strategies to advocate for continued funding for breast cancer screening
New Mexico	Support policy changes to help cancer survivors
New York	Fund professional education programs related to cancer treatment and care
	Support/fund quality improvement collaboratives to ensure providers recommend evidence-based, guideline-driven cancer screening, diagnostic, and treatment services
	Support tobacco control and education policies
North Dakota	Support cancer screening programs and care for un- and under-insured individuals
Ohio	Advocate for funding for tobacco control (use of settlement money) and research
Rhode Island	Encourage funding of screening
	Support policies related to tobacco control, physical activity in schools, and skin cancer screening
South Carolina	The plan contains a section on health policy and advocacy. The coalition works to ensure that laws, ordinances, and policies protect our citizens from cancer
Tennessee	Promote and support funding for research, nutrition education, screening, colorectal cancer control, the under-insured, and prostate cancer awareness
Texas	Develop or strengthen the infrastructure supporting the delivery of cancer prevention and care services
	Advocate for funding for tobacco control, obesity prevention and control, nutrition and physical activity recommendations, epidemiologic and environmental monitoring and research, survivorship programs to improve quality of life, and infrastructure supporting collection of quality cancer data and delivery of cancer prevention and care
	Promote funding opportunities across the spectrum of cancer research
Utah	Secure the resources needed to execute the plan
	The Utah plan also includes detail on plan implementation, which supports the concept of sustainability: the Utah Cancer Action Network consists of implementation teams. Each team is asked to select at least one objective or strategy to work on over the course of a year. Teams develop an action plan that provides an outline for accomplishing their chosen intervention. As part of the action plan, teams identify which goal, objective, and strategy they will work on, what will be measured, and what activities will need to be completed in order to accomplish the task. A lead person is selected, key partners identified, a timeline is set, and needed resources determined
Virginia	Advocate for education policies regarding skin cancer, an increase in the number of adolescents and young adults that receive the HPV vaccination, improved access to evidenced-based programs for early detection, healthcare policies that promote appropriate use of palliative care
Wisconsin	Strategies and action steps will be prioritized annually for implementation by the Steering Committee through a systematic process using specific, measurable criteria. The resulting priorities will set the direction for the implementation efforts of the state-wide coalition for the following year
Wyoming	Advocacy efforts are highlighted as steps to sustain the work of the coalition

### Case examples of sustainability

Details on how sustainability is addressed in several of plans are noted:

∙ California: sustainability related to health disparities includes recommendations to provide capacity building, technical assistance, and resources to sustain local efforts; expand federal and state funding for services related to reducing disparities, especially in areas identified as gaps; assist local coalitions in identifying outside private funding sources.
  For site-specific cancers, the California plan notes (prostate cancer) support funding for research to identify better screening tools and develop treatment options; (breast cancer) support research and grants for clinical trials, state and federal funding for access to screening and diagnosis, private funding and grants for uninsured and under-insured people; (cervical cancer) develop and promote clinical standards and professional education materials that promote follow-up and treatment for abnormal screening tests and support funding for state and federally funded programs.
  Advocacy is identified as a tool that can help educate public, healthcare professionals, and policy makers to garner support for funding; participate in media advocacy efforts.
∙ Connecticut: the Connecticut plan notes, “This 4-year plan does not address implementation funding issues directly. Focusing on policy, systems, and environmental (PSE) changes provides a sustainable high-impact approach to health improvement efforts. We charge all organizations to take an active role, working collaboratively to leverage support for implementation and to move forward, using this blueprint as a consensus-based guide for resource allocation.”
∙ District of Columbia: sustainability is a cross-cutting theme through the D.C. plan with references to identifying and seeking funding.
∙ Massachusetts: the plan notes the importance of developing and sustaining collaborations to reduce cancer-related health disparities and promote health equity; creating and sustaining a cancer policy and legislative agenda that supports projects across the continuum; and creating and sustaining environments that support prevention.

### Texas case study

“Organizations, institutions, community leaders, planners, coalition members, cancer survivors, and family and friends affected by cancer from across the state have come together to help develop and implement the *Texas Cancer Plan*, the state-wide blueprint for cancer prevention and control in Texas^2^. The plan addresses the entire spectrum, from cancer research, prevention, and control areas including risk reduction, early detection, and screening, to diagnosis, treatment, palliation, quality of life, survivorship, research, and commercialization. Identifying the challenges and issues that affect our state, the plan presents a set of goals, objectives, and strategic actions to help inform and guide communities and partners in the fight against cancer” (The Texas Cancer Prevention Plan).

The plan was developed by the Cancer Prevention and Research Institute of Texas (CPRIT) with state and CDC funding, which supported the engagement of a professional facilitator and also covered meeting costs. While CPRIT has statutory responsibility for facilitating the development of the CCC plan and supporting its implementation, the overall outcome and success of the plan depend on the cooperation, collaboration, and resources of the many stakeholders throughout the state (p. 2).

The planning process benefited from the involvement of several planning group members who had been involved in the development of previous Texas plans as part of the Cancer Alliance of Texas, a state coalition funded through the CDC. Members without previous CCC prevention planning experience in Texas contributed valuable planning and technical expertise into the process.

In addition to the work of the planning group members, feedback, and comments on the draft content of the Texas plan was sought from community stakeholders including state legislators, members of government-based organizations, industry, and educational and cancer advocacy groups. This important step facilitated meaningful stakeholder input and community buy-in, which in essence lead to community capacity building.

The principles outlined in the plan’s introduction include:

1. Defining measurable and realistic targets based on review of a baseline and trend data for cancer prevention and control key measures, with consideration of factors such as available resources, barriers, and capacity for implementation of strategic actions.
2. Developing or strengthening infrastructure supporting the delivery of cancer prevention and care services.
3. Advocating for funding for tobacco control, obesity prevention and control, nutrition and physical activity recommendations, epidemiologic and environmental monitoring and research, survivorship programs to improve quality of life, and infrastructure supporting collection of quality cancer data and delivery of cancer prevention and care.
4. Promoting funding opportunities across the spectrum of cancer research.

Principles one and two support the construct of capacity building, while three and four support sustainability.

The Texas plan is organized around six priority areas, within which 16 goals are outlined. Experts in the field of cancer prevention and control and public health selected the priority areas based upon review of cancer trends, health disparities, and available evidence-based strategies. If implemented in systematic and comprehensive ways, these priority areas will have a significant impact on the human and economic cancer burden in Texas. Capacity building and/or sustainability are either explicitly or implicitly mentioned in most priority areas and their supporting goals and action statements. The priority areas are:

1. primary prevention and risk reduction (goals 1–6);
2. screening and early detection (goals 7–10);
3. diagnosis, treatment, and palliation (goal 11);
4. quality of life and survivorship (goal 12);
5. infrastructure (goal 13);
6. research and commercialization (goals 14–16).

In terms of capacity building, the Texas plan is effective in engaging community partners at multiple levels. Training and communications strategies (key capacity-building tools) are identified in many of the plan’s goals. As an example, goal 1 of primary prevention is to reduce the incidence and mortality from lung cancer and other tobacco-related cancers. This goal includes several capacity-building actions: improving professional knowledge, practice behaviors, and systems support; development of state-wide messaging campaigns; and implementation of evidence-based strategies for tobacco control.

While not a part of the plan document, CPRIT, which administers $3 billion in funding for cancer research and prevention programs in Texas, requires organizations submitting proposals for funding to detail which goals and objectives their proposals support. The Cancer Alliance of Texas also requires organizations seeking support letters for CPRIT proposals to identify explicitly how their proposals address the plan goals and objectives. These processes build both capacity and bridges to sustainability.

Advocacy activities and funding strategies are found throughout the plan. These approaches are intended to support efforts to sustain the plan’s proposed activities. The Texas Department of State Health Services (TXDSHS), which administers the CDC funding for cancer control in Texas, also supports regional cancer coalitions in order to further achieving the Texas plan’s objectives. The Cancer Alliance of Texas is collaborating with CPRIT and TXDSHS in an evaluation and assessment of the plan due for publication in mid-2015.

The next Texas CCC plan will be developed in 2016. Groundwork is already being laid to support both capacity building and sustainability through activities to reach broader audiences, link organizations engaged in advocacy activities, engage more businesses, and more actively engage social media.

## Discussion

This review adds to a growing literature assessing the content of CCC plans and their incorporation of evidence-based cancer prevention and control practices (15, 16). Although capacity building and sustainability are critical evidence-based building blocks for achieving public health change and improving cancer prevention and control, this review indicates that there is little explicit attention to these concepts in the majority of written materials presented in state CCC plans. As in other areas, there is often a mismatch between research and practice where what is known is often not translated into practice (17).

While community capacity building is not explicitly discussed in the majority of the plans, it is implicit throughout most of the documents through goals, objectives, and strategies that relate to activities including the need for partner organizations to work collaboratively. Training, a vital element in community capacity building (both for health professionals, public policy experts, patients, and caregivers), is mentioned in multiple plans. As an example, the Maryland plan addresses training of professionals, patients, and caregivers in several sections of the document. The New Mexico plan similarly addresses the need for medical professional training, especially in cultural competencies related to tribal communities and older populations.

Public policy or advocacy is addressed in almost all of the plans, either as a stand-alone section or as objectives or strategies supporting goals. Efforts related to public policy or advocacy generally relate to capacity building as well as sustainability and are undertaken by non-governmental CCC coalition members and/or member organizations.

Some plans are including a section outlining what can be done by groups or individuals such as professional organizations, employers, schools and universities, faith based organizations, physicians, legislators, and citizens. At a macro level, this might be considered capacity building, although there is little in the way of how these lists might be put into action beyond dissemination of the plans.

In considering sustainability, ongoing funding is usually a paramount factor, yet the 2005 Review of Comprehensive Cancer Control Plans notes, “Few plans identify funding sources. A few plans describe specific committees or groups charged with identifying or securing funding” (3). Several state CCC organizations have established their own or subsidiary 501c3 foundations that allow them to accept the CDC funding and to pursue other donations and grants to support programmatic efforts. Examples are Georgia, Virginia, Arkansas, Maine, and Kentucky. While the majority of plans include strategies indicating that funding will be sought, there are few details as to how these strategies will be carried out, which agencies will take the lead, which agencies will be involved, or how funds will be managed and administered. It might be assumed that the consortiums intend for funding initiatives activities to be handled by each individual agency, but this is not stated. Since these details are not specified, both funding accountability and monitoring are left open-ended.

In plans including objectives or strategies for funding or support of funding, the majority of these objectives or strategies are included in, but not necessarily limited to, the tobacco prevention sections of the CCC plans. A conclusion might logically be drawn that committees/work groups made up of individuals with extensive long-term experience in tobacco control issues may have more experience in strategies and activities that support funding for public policy and advocacy. Alternatively, it is likely state Tobacco Funds are frequently one of the few sources available to the CCCs other than the CDC funds.

As CCC coalitions undertake CCC plan revisions and develop new CCC plans, they might be encouraged to include sections that clearly outline both capacity building and sustainability, as well as specifics regarding roles, responsibilities, and methods for monitoring and evaluation adherence to evidence-based principles (16–19).

In recent years, we have seen a proliferation of dissemination and implementation models that are just beginning to be incorporated into cancer prevention and control research. Models such as the RE-AIM model (20, 21) or the interactive systems framework (22) may prove helpful in the development of future plans.

### Limitations

This report is limited to a content review of current state plans. An initial limitation is that there may have been more current plans that the investigators were able to identify due to posting requirements. For identified plans, community capacity and sustainability constructs may have been addressed in both the planning process and as part of the implementation process, although absent in the plans themselves. Yet, we would argue that giving salience to these concepts in the CCC plan itself is important for establishing credibility and legitimacy to public health processes for building community capacity and achieving sustainability.

Limitations reported in the 2005 CCC plans: a content review (3) can essentially be repeated in this report, and should be addressed in future plan development:

1. “Currently, there is no prescribed or standardized format for CCC plans. Thus, plans vary in organizational structure, length, and level of detail.” Attempts to identify and examine how capacity and sustainability were addressed remain difficult since there is not a standard method or metric available for states to use in addressing these constructs or to objectively assess how well these constructs are dealt with on a state-by-state basis. Because of their critical importance in effective planning, a framework or outline for how and where to address capacity building and sustainability, as well as how to evaluate them, would be useful to state CCC coalitions.
2. Similarly, the use of key word and nomenclature searches to scan plans is helpful, but limiting. As noted in the 2005 review, because many plans use words that differ from those used in a “key word scan,” activities related to capacity building and sustainability might have been missed, even with the added step of a time-consuming read-through of each plan.

## Recommendations for Future Plan Development

Based on this review, the following recommendations are made. These are segmented into recommendations for the Center for Disease Control and Prevention (CDC) Division of Cancer Prevention and Control and for state cancer control consortiums.

For CDC Division of Cancer: Prevention and Control, NCCPC

∙ Provide clear and easy to understand definitions of both community capacity building and sustainability for collaboratives to use in their planning process;
∙ Review the Guidance for CCC Planning Volume 1: guidelines and models and language to better assist states in addressing sustainability and capacity issues in their plans;
∙ Address community capacity building and sustainability constructs through orientations, training, and tools that can be used by CCC Coalitions (e.g., plan developers);
∙ Promote best practices by recognizing and highlighting those plans that are specific in addressing community capacity building and sustainability;
∙ Offer training (online and/or at meetings) related to community capacity building and sustainability related to concrete methods to include these in the planning process;
∙ Encourage use of planning templates by states in order to save time and better leverage resources. These templates, which can be modeled from examples of plans or portions of plans that might be considered “best practices,” can address a variety of cross cutting issues including capacity and sustainability. Use of templates can free state planners from developing processes and formats, and allow them to spend more time on the important content of their plans.

For state CCC coalitions:

∙ Stipulate that each group that works on a section or sections of the CCC plan should include specific steps for how capacity building and sustainability will be addressed. Or address capacity building and sustainability as stand-alone cross-cutting topics within the plan.
∙ Review other state plans to see how both sustainability and capacity building are addressed, and replicate quality processes as appropriate.
∙ Include experts in capacity building and sustainability processes in the planning process (either in an *ad hoc* manner, or as members of the CCC coalition).
∙ Utilize tools and resources available from CDC related to capacity building and sustainability.
∙ Request training and technical support for development of capacity building and sustainability strategies within plans.

## Concluding Remarks

We are hopeful that there may be greater attention to these key concepts as recent CDC funding opportunities have encouraged the development of sustainability efforts (23). Further, strategic planning usually involves fundamental choices about the mission, goals, or vision an organization will pursue; audiences/clients to be served; the organization’s role in the community; programing, services, or products to be offered; and resources needed to succeed (24). These elements are critical to organizational or agency plans as well as coalition plans intended to better serve the community.

The ability of a plan to be carried out is reliant on the capacity of the organization or organizations responsible for implementation, and also the availability of resources to sustain the work of the plan. CCC plans must address both community capacity building and sustainability in a concrete and realistic manner to assure the success of the important work being undertaken by cancer control and prevention agencies and by associations engaged in the planning process.

## Author Contributions

MO and CM conceived the original study, advised on overall research design, and advised on analysis plan and write-up. BS conducted the content review and drafted the manuscript. DVD contributed to the case study and provided a critical review of drafts.

## Conflict of Interest Statement

The authors declare that the research was conducted in the absence of any commercial or financial relationships that could be construed as a potential conflict of interest.

## Footnotes

^1^http://cancercontrolplanet.cancer.gov/index.html

^2^http://www.cprit.state.tx.us/about-cprit/texas-cancer-plan/
